# Optimizing the Predictive Ability of Machine Learning Methods for Landslide Susceptibility Mapping Using SMOTE for Lishui City in Zhejiang Province, China

**DOI:** 10.3390/ijerph16030368

**Published:** 2019-01-28

**Authors:** Yumiao Wang, Xueling Wu, Zhangjian Chen, Fu Ren, Luwei Feng, Qingyun Du

**Affiliations:** 1 School of Resources and Environmental Science, Wuhan University, Wuhan 430079, China; wymfrank@whu.edu.cn (Y.W.); renfu@whu.edu.cn (F.R.); lwfeng@whu.edu.cn (L.F.); 2 Institute of Geophysics and Geomatics, China University of Geosciences, Wuhan 430074, China; snowforesting@163.com; 3 Zhejiang Academy of Surveying and Mapping, Hangzhou 310012, China; chen_cehui@163.com; 4 Key Laboratory of GIS, Ministry of Education, Wuhan University, Wuhan 430079, China; 5 Key Laboratory of Digital Mapping and Land Information Application Engineering, National Administration of Surveying, Mapping and Geoinformation, Wuhan University, Wuhan 430079, China; 6 Collaborative Innovation Center of Geospatial Technology, Wuhan University, Wuhan 430079, China

**Keywords:** landslide susceptibility, Lishui City, machine learning, SMOTE, slope units, neighborhood rough set theory

## Abstract

The main goal of this study was to use the synthetic minority oversampling technique (SMOTE) to expand the quantity of landslide samples for machine learning methods (i.e., support vector machine (SVM), logistic regression (LR), artificial neural network (ANN), and random forest (RF)) to produce high-quality landslide susceptibility maps for Lishui City in Zhejiang Province, China. Landslide-related factors were extracted from topographic maps, geological maps, and satellite images. Twelve factors were selected as independent variables using correlation coefficient analysis and the neighborhood rough set (NRS) method. In total, 288 soil landslides were mapped using field surveys, historical records, and satellite images. The landslides were randomly divided into two datasets: 70% of all landslides were selected as the original training dataset and 30% were used for validation. Then, SMOTE was employed to generate datasets with sizes ranging from two to thirty times that of the training dataset to establish and compare the four machine learning methods for landslide susceptibility mapping. In addition, we used slope units to subdivide the terrain to determine the landslide susceptibility. Finally, the landslide susceptibility maps were validated using statistical indexes and the area under the curve (AUC). The results indicated that the performances of the four machine learning methods showed different levels of improvement as the sample sizes increased. The RF model exhibited a more substantial improvement (AUC improved by 24.12%) than did the ANN (18.94%), SVM (17.77%), and LR (3.00%) models. Furthermore, the ANN model achieved the highest predictive ability (AUC = 0.98), followed by the RF (AUC = 0.96), SVM (AUC = 0.94), and LR (AUC = 0.79) models. This approach significantly improves the performance of machine learning techniques for landslide susceptibility mapping, thereby providing a better tool for reducing the impacts of landslide disasters.

## 1. Introduction

Landslides, which result in the loss of human life and property, represent some of the most destructive natural disasters in the world [1]. Mountainous areas are especially to landslides, which are controlled by complex geographical environments and human impacts [2,3,4]. China is one of the most landslide-affected countries around the globe [5]. In China, landslides and debris falls occur over 20,000 times a year on average, resulting in more than 1000 casualties, affecting more than 900,000 disaster-stricken people, and causing 2–6 billion yuan of direct economic losses [6]. Zhejiang Province has been particularly impacted by landslides due to its mountainous environment. For example, a serious landslide occurred in Lishui City, Zhejiang Province, on September 28, 2016, destroying 20 residential buildings and causing 27 people to become lost [7]. As a consequence, landslide susceptibility maps, which depict the spatial distribution of the likelihood of a landslide occurring, are vital for mitigating the effects of landslides through appropriate decision making in landslide-prone regions [8].

The effectiveness of landslide susceptibility mapping depends greatly on the modeling methodology adopted [9]. Five main methodologies are used for assessing the landslide susceptibility: qualitative, deterministic, physical, statistical, and machine learning methods. Qualitative methods depend on the knowledge of the researchers and generally possess a moderate accuracy [10]. Deterministic models generally estimate the safety factors in a particular region [11,12]. However, methods based on physical modeling may be more accurate because they use expressions based on universal physical laws. Furthermore, analyses of past landslides may provide useful data that can be used in methods based on physical modeling. Such methods have recently been proposed, and they have performed well in small areas [13,14,15,16,17,18]. In recent decades, numerous statistical methods, such as weights of evidence, index of entropy, frequency ratio, and statistical index models, have been widely used to map the landslide susceptibility and have achieved good results [19,20,21]. However, statistical models focus on data independence, and thus, some environmental factors cannot be fully satisfied due to their spatial correlation or spatial heterogeneity; moreover, these methods do not effectively address the complex relationships amongst the large number of conditioning factors in complicated landslide systems [22].

Recently, with the rapid development of computer technology and the availability of massive geographic and remote sensing datasets, a variety of machine learning methods, e.g., logistic regression (LR), decision tree, random forest (RF), artificial neural network (ANN), and support vector machine (SVM) models [23,24,25,26,27,28], have been proposed for landslide susceptibility mapping. Accordingly, various studies on landslide susceptibility mapping using machine learning methods have been conducted. Pham et al. [1] utilized 730 translational landslides to perform a comparative study of different machine learning methods, namely, SVM, LR, Fisher’s linear discriminant analysis (FLDA), Bayesian networks (BNs), and naïve Bayes (NB) algorithms, for landslide susceptibility assessments. Their results showed that the SVM model exhibited the best performance among the methods studied. Another study was carried out by Chen [29], in which new ensembles of ANN, maximum entropy, and SVM machine learning techniques were introduced; in their study, 213 landslide samples were employed to train the models, the results of which confirmed that ensemble models outperform single-machine models. Zhou et al. [30] applied SVM, ANN, and LR models to landslide susceptibility mapping using a total of 202 landslide samples, and their results showed that the SVM model was ideal for their study area. In another investigation, Tsangaratos and Ilia [31] tested the landslide susceptibility mapping performances of the LR and NB algorithms in Greece with 116 training samples and found that the NB algorithm performed better.

Heakmann et al. [32] stated that the number of samples has a positive influence on the model used for landslide susceptibility mapping and that the effect increases by a certain degree, after which the effect does not change. However, the review of the literature presented above reveals that an insufficient number of training samples have been used in the various machine learning approaches that have been adopted for landslide susceptibility mapping. In other words, machine learning methods require a large number of samples. Moreover, landslides are not regular events; hence, the amount of landslide data (positive samples) is limited, while the quantity of non-landslide data (negative samples) is very large. Some scholars used the pixel-based method to divide the study area, thereby obtaining additional training samples [1,33]; however, this method has a limited ability to represent both the homogeneity and the heterogeneity across a study area. In addition, previous studies discretized continuous landslide-causing factors into several classes for the convenience of analyzing the factors and eliminating redundant data. Unfortunately, there is a lack of consensus on the discretization approach, which can change the data properties and cause information loss.

To resolve these problems, we adopted slope units (SUs) as mapping units. Twenty initial landslide-causing factors were extracted from topographic maps, geological maps, and satellite images and were then resampled by the SUs. The neighborhood rough set (NRS) method was used to remove redundant factors and maintain the original nature of the data. Then, twelve factors, namely, the aspect, profile curvature, elevation, distance to faults, distance to rivers, distance to roads, land use, normalized difference vegetation index (NDVI), slope, engineering geological type, terrain surface texture (TST), and precipitation composite index (PCI), were selected as the final landslide-causing factors. Seventy percent of the landslide locations were randomly selected to construct the training dataset, while the remainder was used for the testing dataset. Then, we applied the synthetic minority oversampling technique (SMOTE) to oversample the training dataset and obtain datasets ranging in size from two to thirty times that of the original training dataset. The review of the literature presented above shows that SVM, LR, ANN, and RF techniques have been widely used in landslide prediction; therefore, in this study, these machine learning methods were applied for landslide susceptibility mapping, and the effects of different training dataset sizes were compared.

## 2. Materials and Methods

### 2.1. Study Area

**Figure 1:**
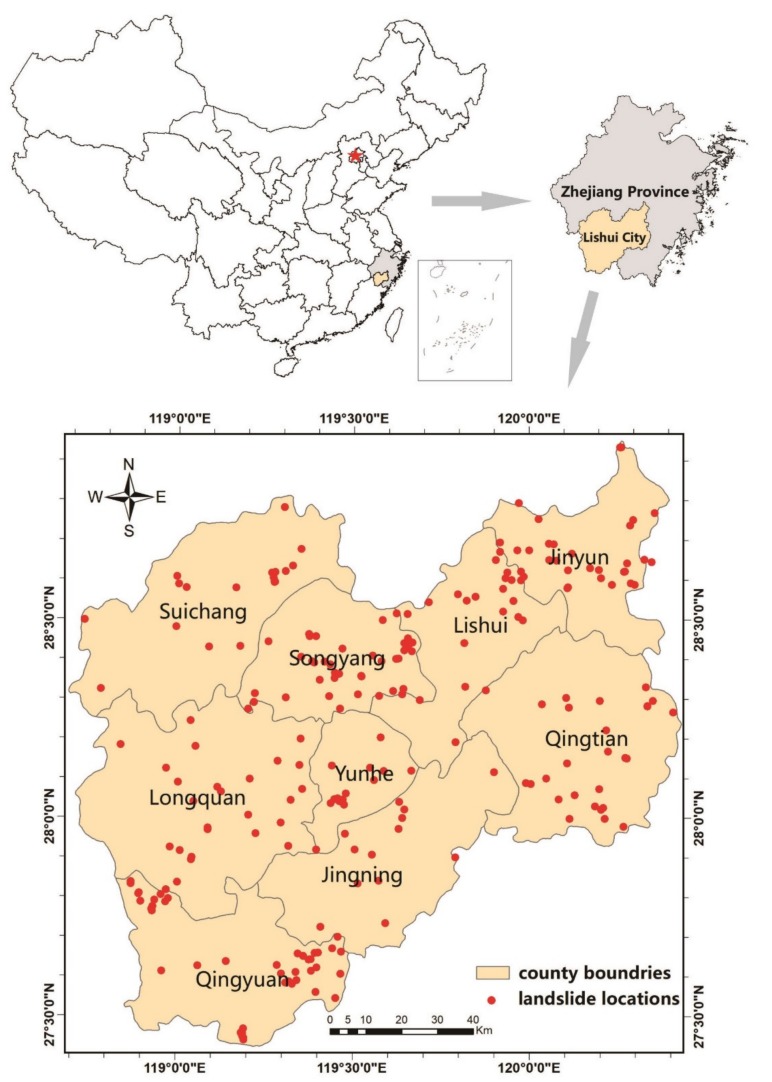
Location map of the study area.

The study area (Lishui City), which is situated at 118°41′–120°26′ east longitude and 27°25′–28°57′ north latitude, is located in the southwest region of Zhejiang Province, China (Figure 1). The study area covers an area of 17,300 km^2^, which is composed of mountainous areas (88.42%); cultivated land (5.25%); and streams, roads, and villages (collectively 6.06%). Lishui City is located within a typical subtropical monsoonal region with warm summers and cold winters. This city has a mean annual temperature of 17.8 °C, while the historical maximum and minimum temperatures are 43.2 °C and −10.7 °C, respectively. The average annual precipitation is 1568.4 mm, which generally decreases from south to north and ranges between 1350 mm and 2200 mm. Eighty percent of the annual rainfall occurs from March to September.

The terrain in the study area is high in the southwest and low in the northeast, and the landscape is dominated by mountains and hills with numerous canyons and narrow mountain basins: these conditions can easily lead to landslides and collapses. The geological structure is dominated by faults; among them, there are two main groups of faults: the Yuyao–Lishui deep fault zone in the northeast and the north-south-oriented Songyang–Pingyang fault. Two major types of lithology outcrop in Lishui City: Mesozoic and Proterozoic [34]. The Mesozoic lithologies, which include Middle Jurassic, Late Jurassic, and Cretaceous strata, consist mainly of pyroclastic rocks and sedimentary rocks; their exposed area accounts for more than 85% of the total area of the city. In comparison, the Proterozoic lithologies are composed primarily of schist, gneiss, and mixed rock series distributed over an area exceeding 1200 km^2^.

The above climatic, geological and environmental characteristics expose Lishui City to frequent geological disasters. Continuous or heavy rain is considered to be the primary cause of natural disasters in the study area. The main types of geological disasters are collapses, landslides, and mudslides, which can range from small- to large-scale hazards [35,36].

### 2.2. Datasets

#### 2.2.1. Landslide Inventory

In this study, landslides were inventoried based on historical records (2012–2017), extensive field surveys, and interpretations of Google Earth satellite images (May 2018). Ultimately, 398 landslide locations were identified. Based on the landslide classification by Cruden and Varnes [37,38], soil slides (288), rock slides (48), falls (42), and flows (20) represent the main types of landslides in the study area. Many scholars have suggested that different types of landslides should be treated separately, but the data on rock slides, falls, and flows are limited, and thus, we consider only soil slides in this work. A total of 288 soil landslide locations are mapped in Figure 1. These landslides are typically small: the smallest landslide area is 200 m^2^, while the largest landslide area is 40,000 m^2^, and the average area is 8605 m^2^. For small landslides, a single point per landslide has been proven to be effective in landslide susceptibility mapping [19,39]; therefore, all landslides in this study are represented by a single dot based on this conception.

#### 2.2.2. Landslide-Causing Factors

The acquisition of information on past landslides is very important [40]. Due to the complex characteristics of the geoenvironment, there are no universal conditioning factors for landslides [25]. Based on previous landslide susceptibility studies, landslide conditioning factors can be approximately divided into two categories: predisposing factors and triggering factors. Predisposing factors refer to the basic geology and topography that provide the necessary environmental conditions for the formation of landslides; these factors are fixed and control the overall pattern of landslides, including their scale and type. In contrast, triggering factors refer to the external causes, such as rainfall, earthquakes, and human activities, which trigger the occurrence of regional landslides.

According to the availability of data within the study area, 15 predisposing factors and 5 triggering factors were considered for modeling. The predisposing factors include the elevation, slope, aspect, curvature, profile curvature, plan curvature, topographic wetness index (TWI), terrain ruggedness index (TRI), TST, distance to roads, distance to rivers, distance to faults, NDVI, land use, and engineering geological type. The triggering factors are composed of the annual precipitation, annual precipitation in the wet season, annual precipitation in the dry season, annual torrential rain days and earthquake influence. Detailed information on these 20 landslide conditioning factors is shown in Table 1, and a brief description of the preparation of each controlling factor is given below.

The elevation, slope degree, slope aspect, surface curvature, profile curvature, plan curvature, TWI, TRI and TST are topographic factors. These factors were extracted from a digital elevation model (DEM) with a resolution of 30 m (http://www.gscloud.cn) originally sourced from the ASTER GDEM V2 (National Aeronautics and Space Administration, Washington District of Columbia, USA) produced by the ASTER sensor, which is located on board the TERRA satellite. The elevation is the attribute of the DEM. The slope degree, slope aspect, surface curvature, profile curvature, and plan curvature were calculated by the raster surface toolbox in ArcGIS Pro 2.1 software (Environmental Systems Research Institute, Redlands, California, USA) [41]. The TWI, TRI, and TST were generated by SAGA 6.1 software (SAGA User Group Association, Hamburg, Germany) [42]. The distance to roads and distance to rivers were produced by the spatial distance analysis tool in ArcGIS based on topographic maps at a scale of 1:50,000. The distance to faults was derived from a geological map at a scale of 1:500,000. The engineering geological type was extracted from engineering geological maps at a scale of 1:500,000 and then reclassified into 10 groups: group 1 (hard intrusive rock), group 2 (hard lava), group 3 (hard metamorphic rock), group 4 (water bodies), group 5 (soft clay), group 6 (hard sedimentary rock), group 7 (hard layered tuff), group 8 (carbonaceous rock), group 9 (red rock), and group 10 (continental soft soil). Satellite images from the Landsat 8 OLI with a resolution of 30 m (https://earthexplorer.usgs.gov) were used to obtain the NDVI and land use data within our research area. The preprocessing for each image included radiometric, atmospheric, and geometric distortion corrections. Subsequently, we processed the images using supervised classification in ENVI 5.1 (Harris Geospatial Solutions, Broomfield, Colorado, USA) with reference to Google Earth. The final land uses are categorized into 8 types: roads, structures, water, planting land, desert and bare land, forest and grass, artificial heap, and house buildings (the classification is in accordance with the Chinese land use survey standard). The NDVI, which was used to reflect the vegetation cover, was calculated using the red and near-infrared bands of the images.

**Figure 2:**
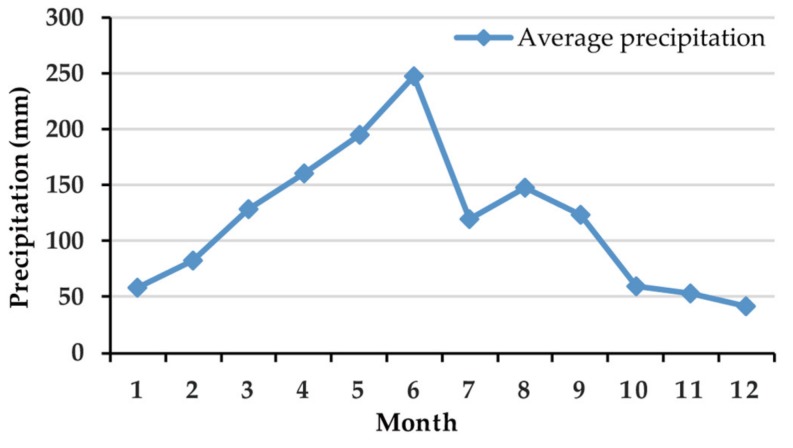
Monthly average precipitation from 2011 to 2017.

Earthquakes and precipitation are considered triggering factors that often accompany the occurrence of landslides [43]. Over the last 40 years (1976–2018), 51 earthquakes have occurred in Lishui City. We obtained information on the location and magnitude of each earthquake from the China Earthquake Networks Center (http://news.ceic.ac.cn) and then used the kernel density method [44] to generate a seismic influence map, which indicates the influence of earthquakes on regional landslides. Precipitation has long been regarded as one of the most important factors influencing landslides. To analyze the influence of rainfall on the occurrence of landslides, 7 years of data (2011–2017) from 279 meteorological stations distributed throughout the study area were collected from the China Meteorological Administration. Rainfall has a high variance among different months; two peak rainfall periods occur in June and August, as shown in Figure 2, in which the precipitation data are summarized to highlight the seasonal trend. We divided the precipitation data into a wet season (April to September) and a dry season (January to March and October to December) based on the intensity and duration of rainfall in the study area to explore its influence on landslides. Additionally, landslides have been proven to be heavily dependent on the short-period rainfall intensity [45]. From the above work, four factors were extracted as rainfall-related conditioning factors: the annual precipitation, annual precipitation in the wet season, annual precipitation in the dry season, and annual torrential rain days (defined as days when the 24-hour rainfall is greater than 50 mm). These factors were computed for each meteorological station, after which they were spatially interpolated throughout the study area using the kriging interpolation method to obtain spatial distribution maps for the four factors.

**Figure 3:**
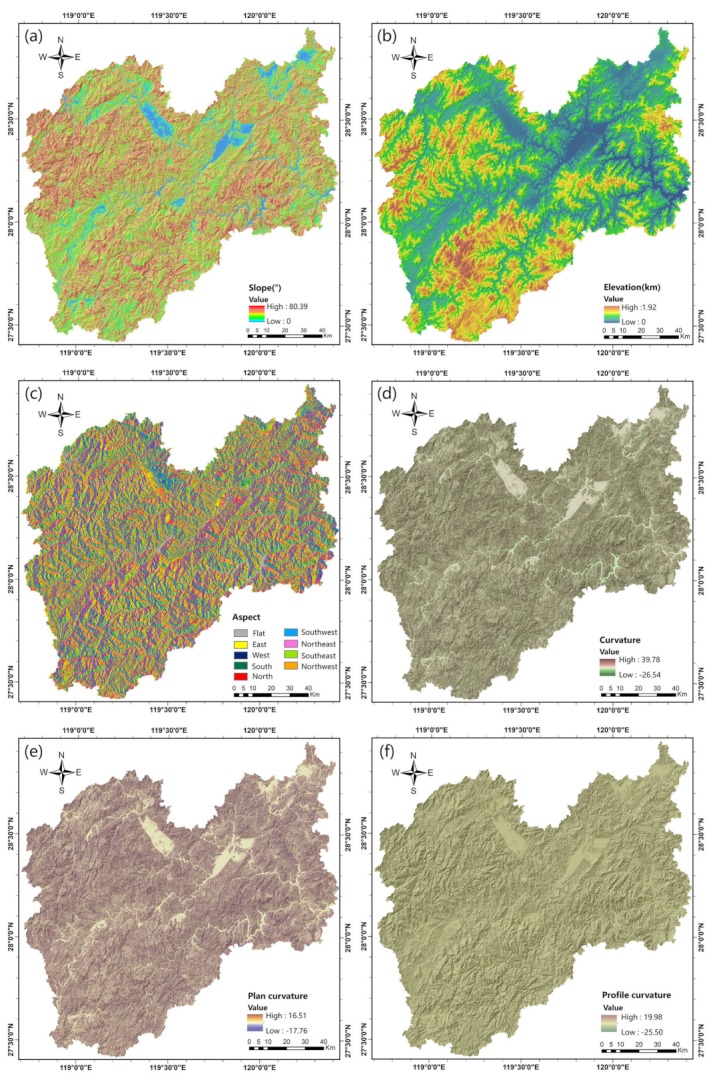

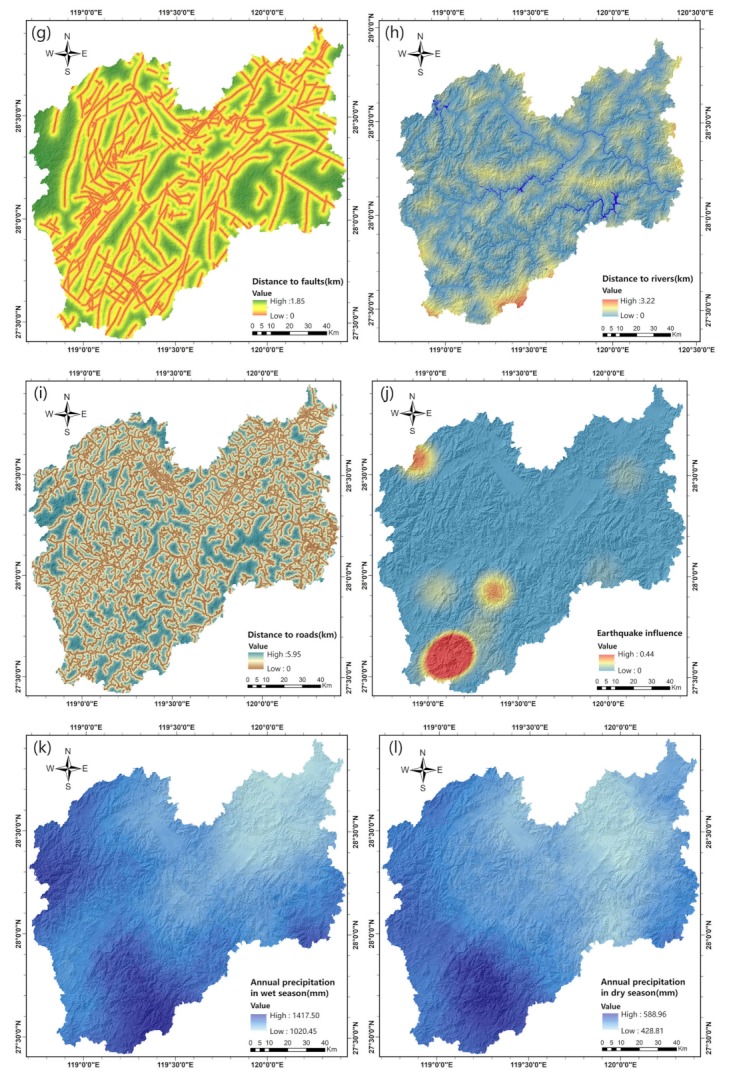

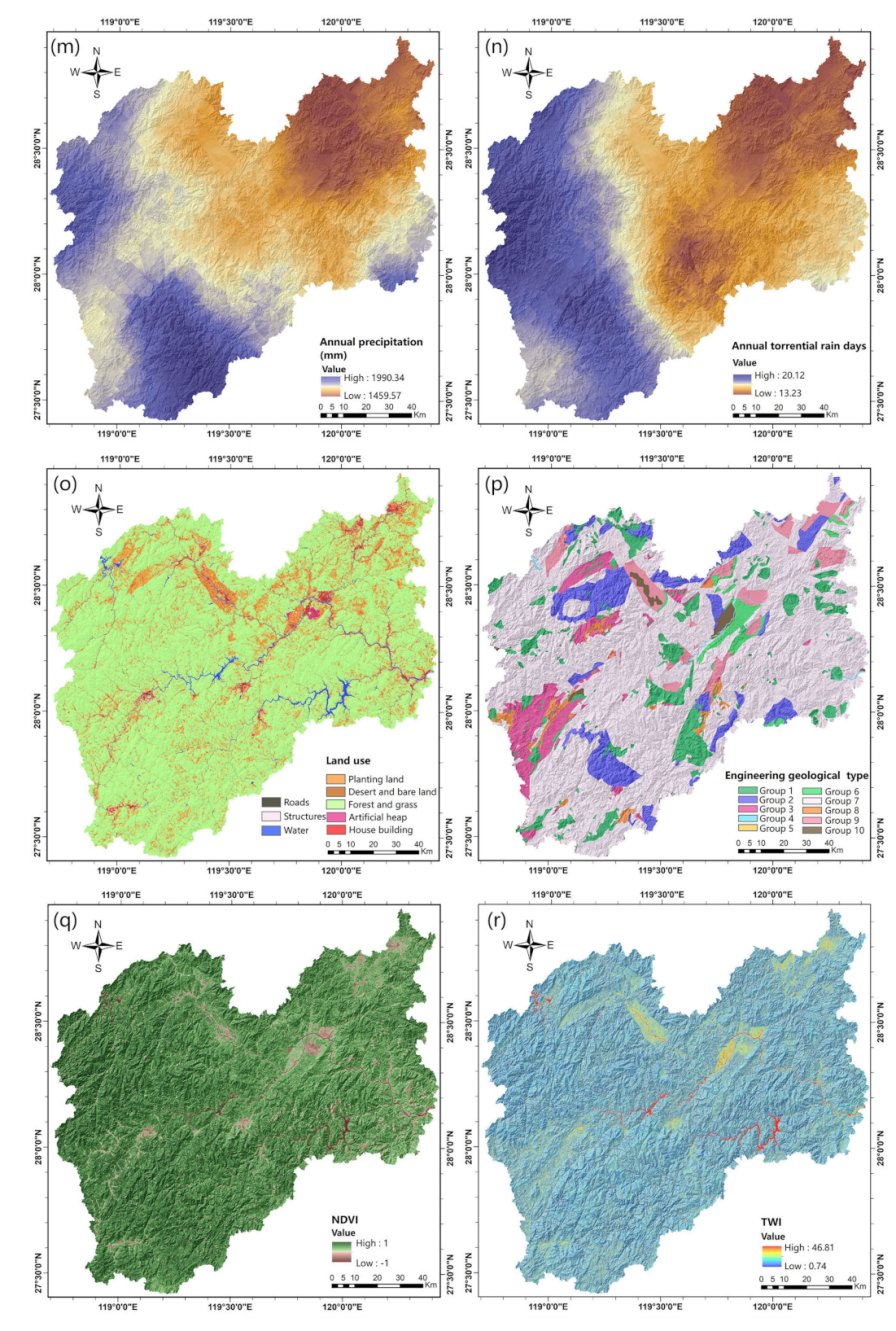

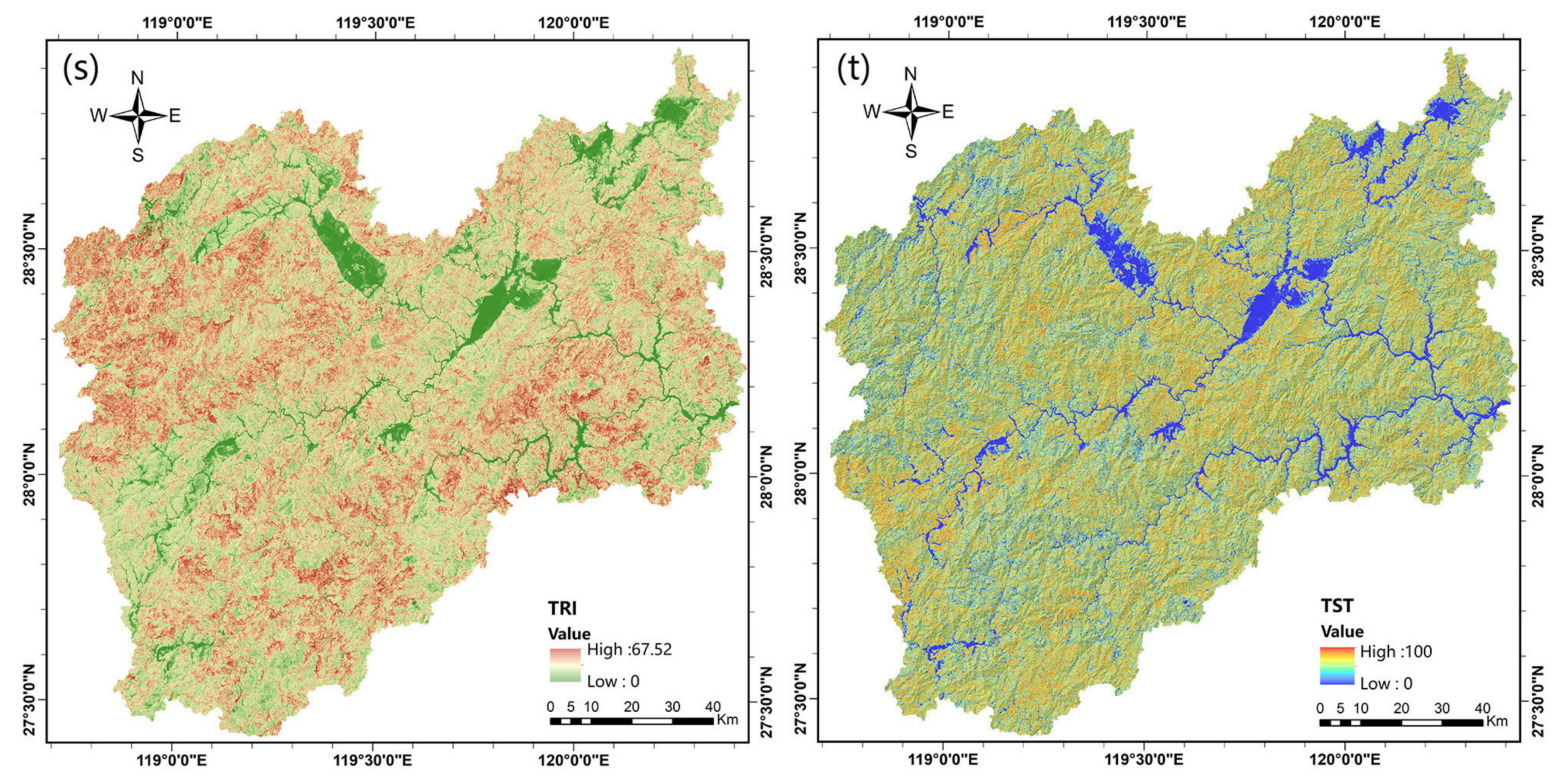
Thematic maps of the landslide-causing factors: (**a**) slope; (**b**) elevation; (**c**) aspect; (**d**) curvature; (**e**) plan curvature elevation; (**f**) profile curvature; (**g**) distance to faults; (**h**) distance to rivers; (**i**) distance to roads; (**j**) earthquake influence; (**k**) annual precipitation in the wet season; (**l**) annual precipitation in the dry season; (**m**) annual precipitation; (**n**) annual torrential rain days; (**o**) land use; (**p**) engineering geological type; (**q**) NDVI; (**r**) TWI; (**s**) TRI; and (**t**) TST.

All the landslide predisposing factor maps were converted into raster format with a resolution of 30 m, which is consistent with the data sources. Previous studies transformed continuous factors (such as the elevation) into discrete factors for convenience [39]. However, this approach may reduce the intrinsic information of the data. In this study, we maintained the characteristics of the predisposing factors. Table 1 shows the variables of each predisposing factor; in total, 17 continuous factors and 3 discrete factors were considered in this study. The spatial distribution of each landslide predisposing factor is presented in Figure 3.

### 2.3. Methodology

**Figure 4:**
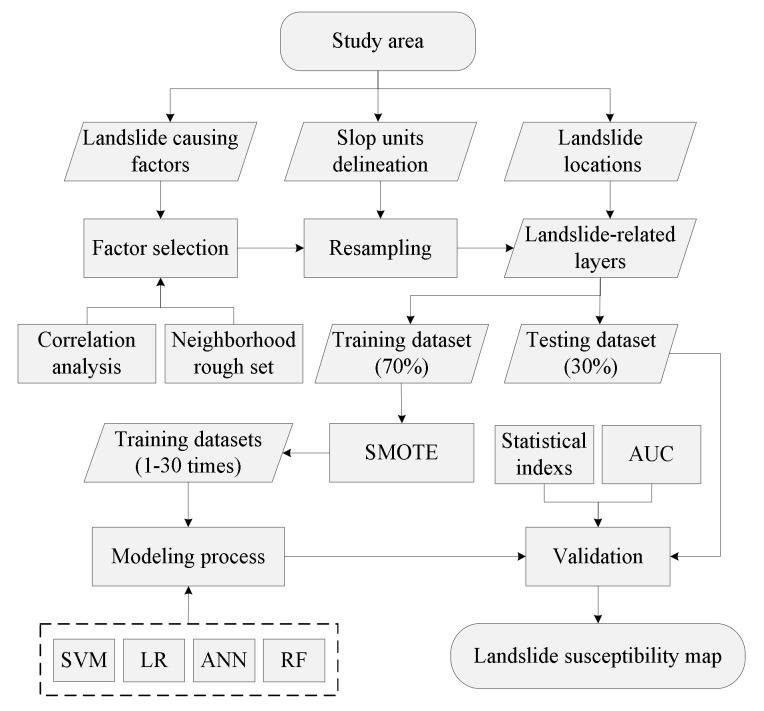
Methodology of the present study.

This paper aims to improve the precision of machine learning models for landslide susceptibility mapping by expanding the sample size. This research was performed using four machine learning models: SVM, LR, ANN, and RF. Each model was constructed with various sample sizes generated using SMOTE to reveal the effect of the sample size on the landslide model accuracy. There are six steps in this process: (1) selecting the landslide conditioning factors; (2) preparing the training and validation datasets; (3) delineating the SUs; (4) implementing machine learning; (5) evaluating and comparing the landslide models; and (6) developing landslide susceptibility maps. Figure 4 shows a flow chart of the methodology utilized in this study.

#### 2.3.1. Selecting the Landslide Conditioning Factors

Landslide susceptibility mapping models depend on the quality of the input data [24] because redundant conditioning factors may produce noise in the modeling process and reduce the predictive capability [46]. Therefore, choosing the optimal conditioning factors for landslide susceptibility mapping is critical. In this study, we first apply Pearson’s correlation coefficient (PCC) and principal component analysis (PCA) to eliminate the factors in highly correlated factor pairs [47]. PCC is a common measure of the degree of linear correlation between two variables that ranges between −1 and 1, where an absolute value of PCC greater than 0.5 indicates a strong correlation. PCA is a popular technique for analyzing and simplifying datasets that uses an orthogonal transformation to convert a set of correlated variables into a set of linearly uncorrelated variables called principal components. Two typical phenomena are encountered in PCA: one is a strong correlation between one factor and multiple other factors without any correlations among the latter and the other is strong correlations among multiple factors. For the first phenomenon, we remove the one factor directly to eliminate the correlation, whereas PCA is used to address the second phenomenon by generating principal components instead of the correlated factors.

Second, the NRS method is used to filter out less important factors. NRS is based on rough set theory (RS), which was proposed by Pawlak [48]. RS is an efficient feature selection method that has been used to eliminate less important landslide-causing factors [49]. The fundamental aspects of the RS-based feature selection technique are briefly introduced as follows.

Let *IS = <U, A, D>* be an information system, where *U = {x_1,_ x_2..._ x_n_}* is a finite set of objects called the universe, *A* is the set of condition attributes, and *D* is the decision attribute.

**Definition** **1.** *X ⊆ U can be approximated using only the information contained in R ⊆ A by constructing the lower approximations $\underline{R}X$ and the upper approximations $\bar{R}X$ of X:*

(1) $$\underline{R}X=\{x\in U|[x]R\subseteq X\}$$

(2) $$\bar{R}X=\{x\in U|[x]R\cap X\neq \emptyset \}$$

*where [x]R denotes the equivalence classes of X. If $\underline{R}X\neq \bar{R}X$, X is called the rough set of U relative to R.*

**Definition** **2.** *If B ⊆ A, the significance of α ∈ B is defined as*

(3) $$\mathrm{Sig}\left(\alpha ,B,D\right)=\gamma_{B}\left(D\right)-\gamma_{\left(B-\alpha \right)}\left(D\right)$$

*where $\gamma_{B}(D)=\frac{\left|\underline{R}X\right|}{\left|U\right|}$ is the dependence degree of D on B.*

If Sig(α,B,D)=0, α is superfluous in B. The RS-based feature selection is designed to find a special subset *B* that represents the entire original dataset with minimal attributes.

However, RS is weak when dealing with continuous data such as the elevation and slope. Previous studies discretized continuous attributes into several classes to offset this deficiency, thereby changing the properties of the data and causing information loss. Thus, this study applies the NRS method proposed by Hu [50] that has been used to reduce numerical and categorical features. The NRS technique is improved by using the neighborhood concept and by redefining both the lower approximations and the upper approximations.

**Definition** **3.** *If U is separated into N equivalence classes denoted as X_1,_ X_2_,…, X_N_ by a decision D, B ⊆ A generates a neighborhood relation N_B_ over U, and the lower and upper approximations of D with respect to the attributes B are defined as*

(4) $$\underline{N_{B}}D=\left\{\underline{N_{B}}X_{1},\underline{N_{B}}X_{2},\ldots ,\underline{N_{B}}X_{N}\right\}$$

(5) $$\bar{N_{B}}D=\left\{\bar{N_{B}}X_{1},\bar{N_{B}}X_{2},\ldots ,\bar{N_{B}}X_{N}\right\}$$

*where $\underline{N_{B}}X_{i}=\left\{{x|\delta }_{B}\left(x\right)\subseteq X_{i}\right\}$, $\bar{N_{B}}X_{i}=\left\{{x|\delta }_{B}\left(x\right)\cap X_{i}\neq \emptyset \right\}$, i = 1, 2, ⋯, N. $\delta_{B}(x_{i})$ is the neighborhood of x_i_ in feature space B, and it can be computed using a distance function. More details on the NRS method can be found in Hu [50].*

#### 2.3.2. Preparation of the Training and Validation Datasets

Landslide susceptibility mapping with data mining methods can be considered a typical binary classification [51]. The most important problem is ensuring that there is a sufficient number of valid samples, including landslide and non-landslide data. Especially with machine learning methods, adequate data are needed to ensure a high performance. However, many studies that applied machine learning methods in landslide susceptibility mapping used only dozens or hundreds of sample data points and therefore may not have demonstrated the full power of machine learning. As a rule, relatively few landslide samples are used because landslides are relatively rare, whereas non-landslide samples are much more numerous than landslide samples and are more easily collected. The use of additional non-landslide samples could expand the data size for machine learning, but it will cause a sample imbalance problem, thereby biasing the classifier towards the non-landslide data and negatively affecting its performance. Based on the work performed by previous researchers [52,53,54], a balanced (1:1) positive (landslide) and negative (non-landslide) dataset has been proposed. Therefore, this article applies SMOTE to oversample sufficient landslide data to match the number of non-landslide samples.

SMOTE is an oversampling technique proposed by Chawla in 2002 [55]. The main idea of this method is to use K-nearest neighbors and linear interpolation to artificially insert new samples according to certain rules between two closely related samples to increase the number of samples and balance the dataset. SMOTE operates in a “feature space” rather than a “geographic space” and is given a specified oversample ratio of the original minority data size *α*. For each sample from the minority class denoted by *x*_0_, its K-nearest neighbors are filtered by the smallest Euclidean distance from the feature space of the original sample, and one of them is randomly chosen (*x_r_*), where K is a manually input hyperparameter. The new synthetic SMOTE sample is defined as

(6) $$x_{s}=x_{0}+\delta \cdot x_{r}$$

where *δ* ∈ [0,1] is a random number that is different for each SMOTE sample. This procedure is repeated until the *α* ratio is met.

In this study, the landslide inventory was randomly divided into two subsets: 70% of the landslide locations (202) were used to train the models and 30% of the landslides (86) were used to validate the constructed models. We used SMOTE to oversample the original training landslide datasets while setting α as an integer array ranging from 2 to 30 and K as 20. Consequently, 30 training landslide datasets (the first training dataset is the original training dataset) were produced with sizes ranging from 202 to 6060. The same number of non-landslide datasets, which were generated randomly from landslide-free areas, was added to the testing landslide dataset and each training landslide dataset. Finally, the training dataset and testing dataset were prepared. In particular, the landslide data in the first training dataset and testing dataset were real samples, and the landslide data in the other 29 training datasets were generated by SMOTE. This means that samples in the first training dataset are all real, and this dataset can function as a control group to compare the benefits for other training datasets to determine if the improvements in the performances of the machine learning methods using SMOTE are significant.

#### 2.3.3. Slope Unit Delineation based on Terrain Curvature

SUs represent the region of space under the constraints of homogeneous slope aspect and steepness distributions [56,57] and can be bound by drainage and divide lines based on geomorphological characteristics. The SU boasts a better geomorphological representativeness than the grid and performs efficiently in landslide susceptibility mapping. In the previous literature, the hydrology analysis-based method has been most commonly employed for the division of SUs. This SU partitioning method segments a watershed based on the elevation and the reversal of the elevation to extract ridge lines and valley lines, after which these terrain feature lines are overlain [58,59]. This method provides an automatic method for delineating SUs by using the hydrology tool in ArcGIS.

However, this approach suffers from a diminished capacity in intermountain basins or large open valleys [60]. Yan Ge [61] improved this method by using the curvature instead of the elevation. First, the DEM is smoothed by an appropriate radius to alleviate intricate changes in partial ground resulting from slope erosion; then, the average curvature of each grid cell in the smoothed DEM is obtained. Second, the watershed can eventually be calculated based on the flow direction and sink by assuming the average curvature as the elevation, obtaining the flow direction and extracting the sink; topologically, the outline of the watershed polygon is the ridge line. Third, to detect the valley line, the curvature data must be reversed—high-curvature values are reversed to low values, and low-curvature values are reversed to high values—after which the second step is repeated with the reversed curvature data. Finally, the ridge lines and valley lines are combined, and the SUs can be obtained.

**Figure 5:**
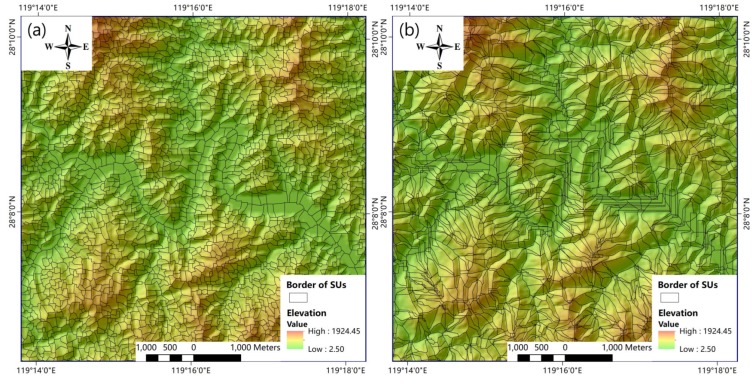
Comparison of the effects of slope units (SUs) using two methods in a certain section of Lishui City: (**a**) improved method and (**b**) hydrology analysis-based method.

Using ArcGIS software, we obtained two types of SUs via the hydrology analysis-based method and the improved method separately. However, manual editing still had to be performed. Tiny and fragmented units needed to be merged into nearby SUs, and the polygons of anomalous SUs were reshaped based on the terrain. Finally, the study area was divided into 438,609 and 914,475 SUs with the hydrology analysis-based method and the improved method, respectively. Figure 5 shows sections of both types of SUs from the same area. The SUs from the improved method are more regular and fit the local terrain better than those from the hydrology analysis-based method. Many anomalously small and long SUs were generated by the traditional method, especially on flat ground, whereas the new method presented better results. Therefore, we adopted the SUs from the improved method in this study. The size of the smallest SU is 1000 m^2^, while size of the largest SU is 538,892 m^2^; the average area is 18,780 m^2^. These SUs are small enough to capture the spatial characteristics of landslides and large enough to reduce the computational complexity. Subsequently, all values of the landslide conditioning factors were calculated for each SU from the raster layers. The average values among all the grids in an SU represent the values of the corresponding continuous factors and the mode for the corresponding categorical factors.

#### 2.3.4. Support Vector Machine (SVM)

The SVM, which was first proposed by Vapnik [62], is a very common supervised machine learning algorithm. SVM classifiers can use fewer samples to achieve a better classification result than other algorithms. There are generally three types of SVM classifiers based on the number of objects being classified: the one-class SVM, two-class SVM, and multiclass SVM [27,28]. In this paper, the two-class SVM is applied to distinguish between landslide and non-landslide areas.

**Figure 6:**
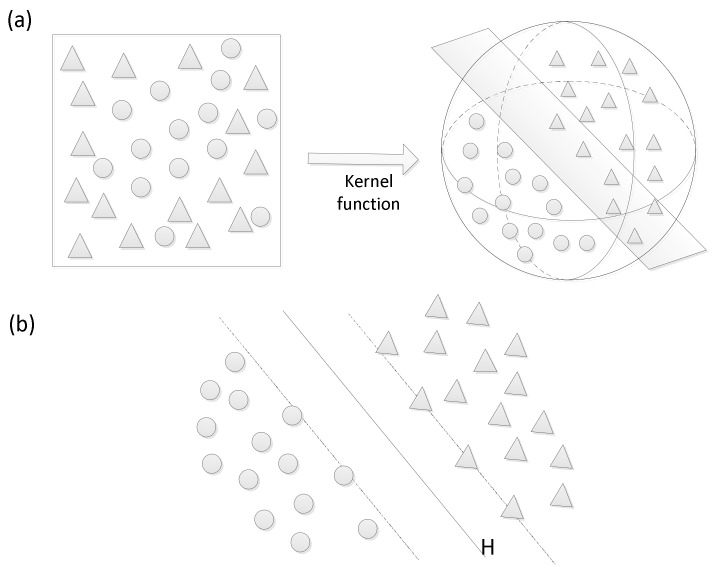
Explanation of the support vector machine (SVM principles): (**a**) the kernel function and (**b**) the optimal hyperplane.

There are two main principles of the two-class SVM [28]. The first is to use a kernel function to map samples from the original space to a higher-dimensional feature space and the second is to find an optimal classification hyperplane used to separate two classes by the maximum gap. Figure 6a shows the kernel function, which makes messy samples linearly separable. In Figure 6b, H denotes the optimal hyperplane that discriminates between the two types of samples. The samples along the dotted borders, along which the distance to these support vectors from the optimal hyperplane reaches a maximum, are called support vectors. In this study, we used Python’s Scikit-learn library [63] to construct and train the model. We adopted one of the most popular kernel functions called the radial basis function (RBF), and the other parameters are assigned the default values.

#### 2.3.5. Logistic Regression (LR)

LR is a nonlinear multivariate statistical model that has been widely applied in landslide susceptibility mapping [1,64,65]. LR has the advantage of determining the likelihood of landslide occurrence based on the relationships between binary outcome variables (landslide or non-landslide) and categorical or continuous predictor variables such as the elevation, aspect, and NDVI. The LR method has the following form [66].

(7) $$\mathrm{logit}\left(y\right)=\beta_{0}+\beta_{1}x_{1}+\beta_{2}x_{2}+\ldots +\beta_{i}x_{i}+e$$

where *y* is the dependent variable, *x_i_* is the i-th explanatory variable, *β*_0_ is a constant, *β_i_* is the i-th regression coefficient. and *e* is the error. The probability (*p*) of the occurrence of *y* is

(8) $$p=\frac{exp^{\left(\beta_{0}+\beta_{1}x_{1}+\beta_{2}x_{2}+\ldots +\beta_{i}x_{i}\right)}}{1+exp^{\left(\beta_{0}+\beta_{1}x_{1}+\beta_{2}x_{2}+\ldots +\beta_{i}x_{i}\right)}}$$

LR uses the sigmoid function to transform the regression value, which may range from −∞ to +∞, to a probability between 0 and 1, where 0.5 is usually used as a threshold to distinguish between landslide and non-landslide samples.

#### 2.3.6. Artificial Neural Network (ANN)

ANNs, which are inspired by biological neural networks [67], have a remarkable ability to determine the meaning and rules of complicated data. They are widely used in many research fields for problems that are too complex to solve using other techniques. ANNs are composed of neurons from three kinds of layers: input, hidden, and output layers. The input layer transmits the original data to hidden layers, and the number of neurons in the input layer depends on the landslide-influencing factors. An ANN contains one or more hidden layers, which process the data through weighted connections and activation functions. The activation function is the key to solving the nonlinear problem; without the activation function, the final result will be a linear change in the original input regardless of the number of hidden layers. The number of hidden layers and their neurons are generally determined by trial-and-error. The output layer accepts the result of the hidden layers and converts the result into the corresponding neurons. The number of neurons in the output layer is equal to the number of types in the classification problem [68].

ANNs perform well with nonlinear issues and, thus, they have been successfully applied in landslide susceptibility assessments [69,70]. Different kinds of learning methods are available: one popular example is a back-propagation algorithm, the main idea of which is to calculate the propagation error, which indicates the difference between the predicted and true values. Then, the weights in this neural network can be updated to achieve an expected goal. This process can be very time consuming due to the complex network structure and the iterative nature of searching for the best performance [49]. We used the Python deep-learning framework Keras [71] to build the ANN: the architecture of the ANN in this work has one input layer, four hidden layers, and one output layer. The input layer has the same number of nodes as the number of landslide conditioning factors. The four hidden layers have 50, 100, 200, and 20 neurons. The hidden layers use the rectified linear unit (ReLU) as their activation function, and the final layer uses a sigmoid activation function to output a landslide probability. Ultimately, we configured the model with the rmsprop optimizer and the binary_crossentropy loss function, and we set the learning rate as 0.001.

#### 2.3.7. Random Forest (RF)

The RF classifier developed by Breiman [72] is another machine learning classification algorithm. This technique is an ensemble learning method that has been widely used in classification. Many decision trees are constructed in an RF model: each decision tree is a classifier and has an output. The RF algorithm integrates all the classification results and specifies the category with the most votes as the final output; furthermore, the RF classifier uses the general technique of bagging, which leads to a better model performance because it decreases the variance in the model and increases the bias [73,74].

There are two types of randomness in an RF. The first is the random selection of training samples; the same training set for each tree will result in the same classification result, which will lose the significance of bagging. The second is randomly selecting the features of the sample. These two types of randomness are very important for the classification performance of the RF. Due to this randomness, an RF can easily avoid overfitting and boasts a good antinoise ability. There are two main parameters in an RF: mtry and ntree. The mtry parameter refers to the number of variables used in each random tree, while ntree refers to the number of trees that the forest contains [75]. In our work, we set mtry to 10 and ntree to 300.

#### 2.3.8. Evaluation and Comparison of Landslide Susceptibility Models

Assessing the performance of a model can be accomplished with different aspects; among them, the fitting and predictive accuracies have long been considered very important features [29]. The fitting accuracy, which measures how the results from a landslide susceptibility model fit the training dataset, can be obtained based on a comparison between the model’s results and the true values in the training dataset. Meanwhile, the predictive accuracy can explain the degree of fit of a landslide model over the testing data. Hence, the performance of a landslide model using training and testing datasets needs to be evaluated to reflect the fitting and predictive capacities, respectively.

In this study, we first applied the accuracy, the kappa index and the area under the receiver operating characteristic (ROC) curve defined as the area under the curve (AUC) to evaluate and compare the performances of the landslide susceptibility models generated from the above four methods using the original training dataset and 29 different sets of training data generated by SMOTE. Therefore, 120 landslide susceptibility models were generated and evaluated to assess not only the performances of the different models but also the influences of different training dataset sizes on each model. The accuracy and kappa index are statistical index-based evaluations that have been commonly used in validating classification models [76]. The accuracy is defined as

(9) $$\mathrm{accuracy}=\frac{TP+TN}{S}\times 100\%$$

where, *TP* is the number of correctly predicted landslide SUs, *TN* is the number of correctly predicted non-landslide SUs, and *S* is the total number of SUs in the study area.

The kappa index is used for testing consistency and can also be used to measure the classification accuracy. The value of the kappa index ranges from −1 to 1; a value >0.4 indicates moderate agreement. The kappa index is defined as

(10) $$\mathrm{kappa}\;\mathrm{index}=\frac{p_{o}-p_{e}}{1-p_{e}}$$

where *p_o_* is the relative observed agreement and *p_e_* is the hypothetical probability of chance agreement.

The ROC has been used to validate the general performance of landslide models in many studies [1,24,33]. The ROC is constructed by plotting statistical index value pairs containing false positive and true positive ratios. The true positive ratio represents the “sensitivity” of a model, while the false positive ratio signifies “1-specificity”. The AUC values range between 0.5 and 1.0—the closer the value is to 1, the better the spatial performance of the landslide susceptibility model [77].

To measure how much the SMOTE method optimized the performance of each model, we computed the percentage of improvement (POI) of each model between the first and 30th training datasets. We define this index as

(11) $$POI=\frac{S_{30}^{x}-S_{1}^{x}}{S_{1}^{x}}\times 100\%$$

where *x* represents an abovementioned evaluation index (the accuracy, kappa index, or AUC), $S_{30}^{x}$ is the performance of a model using the 30th training dataset, while $S_{1}^{x}$ is the performance of a model using the first training dataset.

Second, we chose one model that showed the best performance from each of the four types of methods. Four landslide susceptibility maps were generated using the four different types of models with SU-based probability images. Each SU has a probability value of susceptibility, which ranges from 0.0 to 1.0. The landslide susceptibility maps were reclassified into five classes based on the probability value—very low: 0–0.2; low: 0.2–0.4; moderate: 0.4–0.6; high: 0.6–0.8; and very high: 0.8–1—using an equal interval classification to compare each of the maps. Finally, the class-specific accuracy was computed to examine the spatial distributions and behaviors of the landslide susceptibility maps. The class-specific accuracy, which has often been applied as a measure to evaluate the predictive accuracy in landslide-prone areas in each class of a landslide susceptibility map, is defined as [78]

(12) $$p_{i}=\frac{A_{i}}{B_{i}}\times 100\%$$

where *i* is the susceptibility class (very high, high, moderate, low, or very low) and *A_i_* and *B_i_* are the number of landslide SUs and the total number of SUs, respectively, in the i-th landslide susceptibility zone.

## 3. Results

### 3.1. Elimination of Landslide Affecting Factors

**Figure 7:**
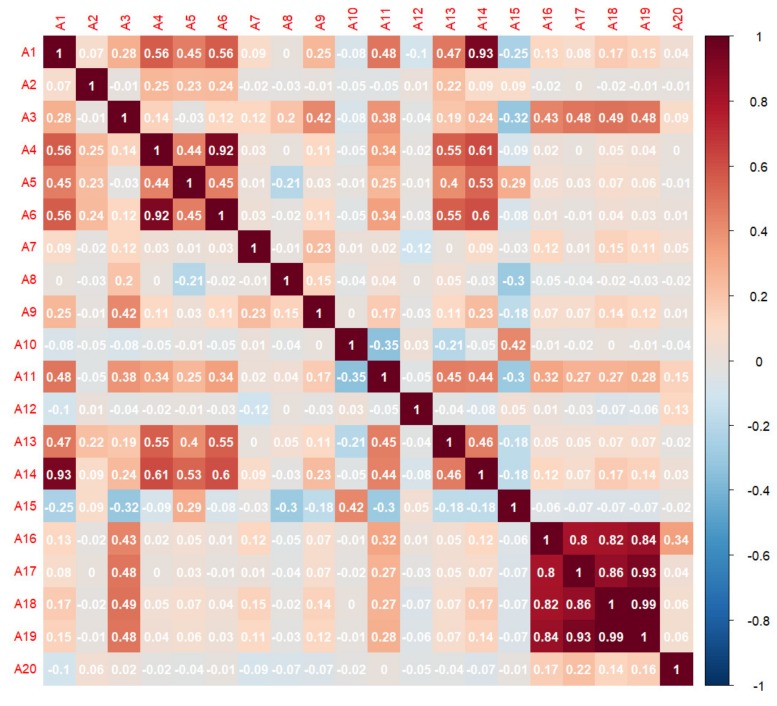
Pearson’s correlation coefficients (PCCs) of the twenty initial conditioning factors. A1: slope; A2: aspect; A3: elevation; A4: curvature; A5: profile curvature; A6: plan curvature; A7: distance to faults; A8: distance to rivers; A9: distance to roads; A10: land use; A11: NDVI; A12: engineering geological type; A13: TST; A14: TRI; A15: TWI; A16: annual torrential rain days; A17: annual precipitation in the dry season; A18: annual precipitation in the wet season; A19: annual precipitation; and A20: earthquake influence.

The correlation coefficients among the twenty initial conditioning factors described in Section 2.2.2 were calculated by PCC, and the results are shown in Figure 7. The correlation coefficients between the curvature and the slope, plan curvature, TST, and TRI are 0.56, 0.92, 0.55, and 0.61, respectively, indicating a strong correlation between the curvature and many other factors. The same situation is observed between the plan curvature and TRI. Therefore, the curvature, plan curvature and TRI were removed from the twenty initial factors to improve the data quality. Furthermore, the correlations between each pair among the annual precipitation, annual precipitation in the wet season, annual precipitation in the dry season, and annual torrential rain days are quite significant. However, directly deleting these four factors is not appropriate because precipitation is an indispensable triggering factor. As noted in Section 3.1, PCA is effective in such cases. Table 2 shows the PCA results. The first principal component (PC1) accounts for approximately 90.434% of the variation in the original dataset; therefore, we chose PC1 as the PCI.

**Figure 8:**
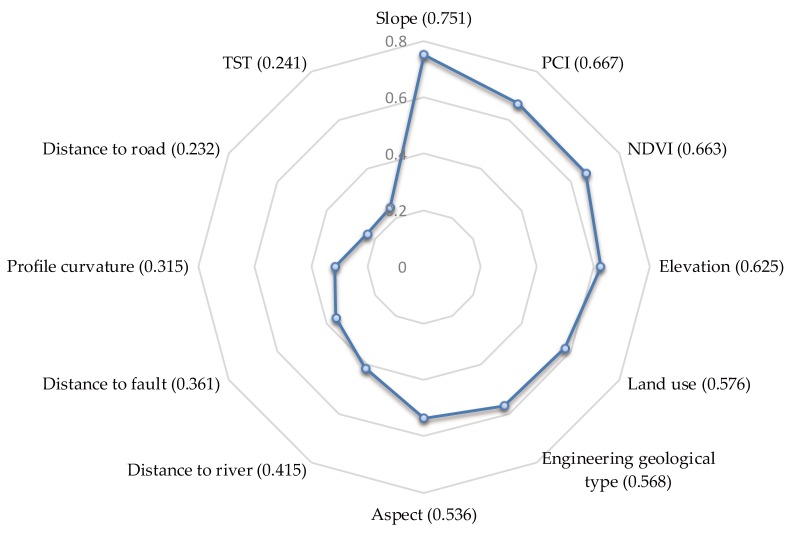
The significance of each of the fourteen factors with respect to landslides.

The remaining fourteen factors were calculated by the NRS method, which is an effective mathematical tool for eliminating superfluous factors in both discrete and continuous data. Two factors (the TWI and earthquake influence) were removed; the significance of each of the remaining factors with respect to a landslide is shown in Figure 8. Therefore, twelve factors were selected as the final landslide affecting factors: the aspect, profile curvature, elevation, distance to faults, distance to rivers, distance to roads, land use, NDVI, slope, engineering geological type, TST, and PCI.

### 3.2. Performances of the Landslide Models

One to thirty multiples of the original training datasets were used to construct the landslide models with 10-fold cross validation, while the testing dataset was utilized to validate the performances of the models. After training and testing the landslide models, the four machine learning models were evaluated according to three criteria: the accuracy, kappa index, and AUC.

**Figure 9:**
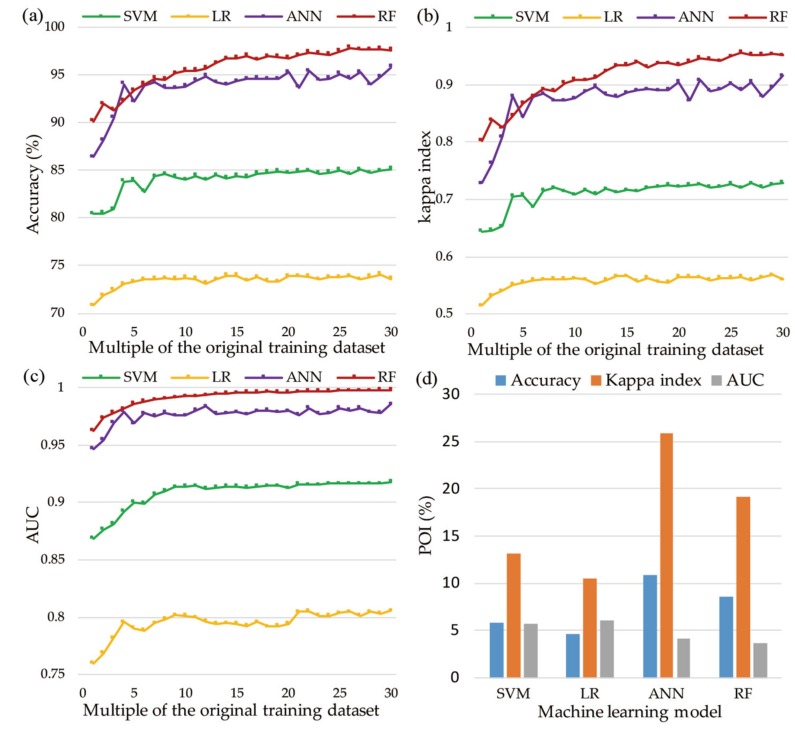
Fitting performances: (**a**) accuracy of each model with different training datasets; (**b**) kappa index of each model with different training datasets; (**c**) area under the curve (AUC) of each model with different training datasets; and (**d**) percentage of improvement (POI) of each model between the first and 30th training datasets.

The performances achieved using the training dataset represent the fitting accuracies of the models. The results in Figure 9 show that the four applied models all exhibited good fitting performances. Each model fluctuated before reaching a multiple of five and stabilized as the size of the training dataset grew. Regarding the accuracy, the RF model performed the best and achieved its highest accuracy of 97.8% at the multiple of 26 (Figure 9a), followed by the ANN model (95.8%), SVM model (85.1%), and LR model (74.1%). The kappa index is another important index for measuring the classification performance. Figure 9b shows that the RF model manifested itself as a perfect classifier (kappa index > 0.8). The ANN and SVM models also achieved a good level (kappa index > 0.6), while the LR model exhibited a moderate performance (kappa index > 0.5). Figure 9c shows that the RF, ANN, and SVM models achieved good landslide susceptibility assessment performances (AUC > 0.85). The RF model performed better than the other models in the vast majority of the training dataset and achieved the highest performance at the multiple of 30 (AUC = 0.998). The ANN model evolved quickly as the training data increased and performed best at the multiple of 30 (AUC = 0.986). The SVM and LR models also both achieved the best fit at the multiple of 30 with AUC values of 0.918 and 0.806, respectively. Figure 9d shows that the fitting performance of each machine learning model showed different levels of improvement as the number of training samples increased. The ANN model showed the most significant improvements, followed by the RF, SVM, and LR models.

**Figure 10:**
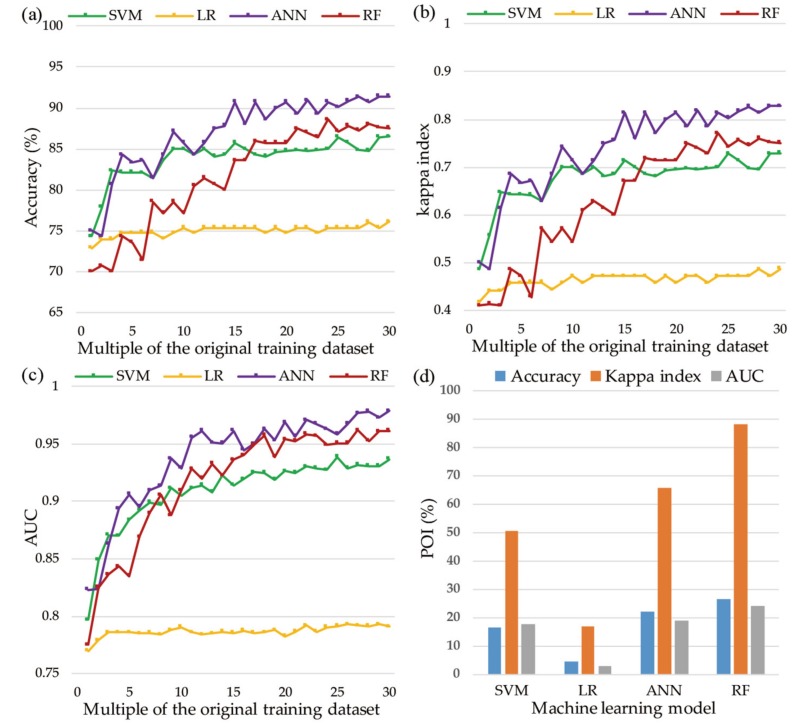
Predictive performances: (**a**) accuracy of each model with different training datasets; (**b**) kappa index of each model with different training datasets; (**c**) AUC of each model with different training datasets; and (**d**) POI of each model between the first and 30th training datasets.

The performances achieved using the testing dataset represent the predictive capacities of the models. The validation and comparison of the landslide models using the testing dataset are presented in Figure 10, which shows that the ANN, RF, and SVM models exhibit marked fluctuations in each criterion before the multiple of 15, while the LR model is slightly smoother. However, as the data size increases, the performances of the landslide models overcome local minima and local maxima, thereby becoming more reliable and accurate. For accuracy, the ANN model performed the best and achieved its highest accuracy of 91.43% at the multiple of 26 (Figure 10a), followed by the RF (88.6%), SVM (86.5%), and LR (76.1%) models. Figure 10b shows that the ANN demonstrated the best classification precision and achieved its highest score at the multiple of 30 (kappa index = 0.83). The RF model achieved the highest kappa index of 0.75, whereas the kappa index values of the SVM and LR models were 0.73 and 0.49, respectively. Figure 10c shows that the ANN, RF, and SVM models performed perfectly in the landslide susceptibility assessment (AUC > 0.8). Moreover, the performances of the three models improved noticeably as the data volume increased, whereas the performance of the LR model improved moderately and then flattened. The ANN model performed the best among the four landslide models, achieving its best level (AUC = 0.98) at the multiple of 30, followed by the RF (AUC = 0.96), SVM (AUC = 0.94), and LR (AUC = 0.79) models. The predictive abilities of the four landslide models improved as the number of training datasets increased, as shown in Figure 10d; the RF model improved the most (AUC increased by 24.12%), and the ANN and SVM models had almost the same improvement (AUC increased by 18.94% and 17.77%, respectively), whereas the LR model improved the least (AUC increased by 3.00%).

### 3.3. Development of Landslide Susceptibility Maps

**Figure 11:**
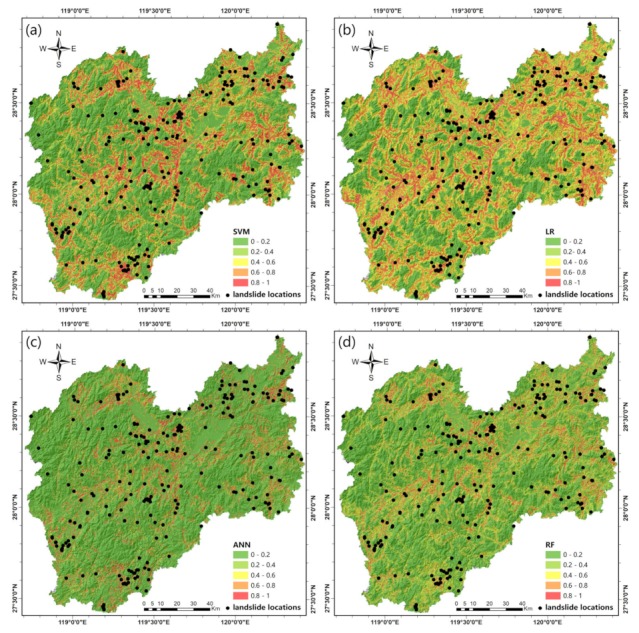
Landslide susceptibility maps: (**a**) SVM model; (**b**) logistic regression (LR) model; (**c**) artificial neural network (ANN) model; (**d**) random forest (RF) model.

We selected the best model from each of the four types of machine learning methods according to their AUC. The four selected methods were used to calculate the landslide probability of each SU in the entire study area. All SUs were reclassified into five classes using an equal interval classification to generate the landslide susceptibility maps (Figure 11); Table 3 shows the class-specific accuracy for each susceptibility class.

According to the spatial distribution of each landslide susceptibility class, the proportion of SUs decreased in the higher landslide susceptibility classes. In the case of the SVM model, the very low susceptibility class accounts for 58.4% of all SUs in the study area, while the low, moderate, high, and very high susceptibility classes account for 13.49%, 10.63%, 10.34%, and 7.13%, respectively. Regarding the LR model, the very low and low susceptibility classes account for 32.24% and 23.06%, respectively, of all SUs, while a total of 21.62% of all SUs belong to the moderate class, and the high and very high susceptibility classes account for 17.38% and 5.70%, respectively, of all SUs in the study area. In the landslide susceptibility map generated by the ANN model, the proportions of the susceptibility classes ranging from very low to very high are 66.39%, 16.03%, 7.89%, 4.53%, and 5.17%, respectively, of all SUs. For the RF model, 59.36% of all SUs in the study area belong to the very low susceptibility class, and 21.21% of all SUs fall into the low susceptibility class, while a total of 10.92% of all SUs are in the moderate susceptibility class, and the high and very high susceptibility classes account for 4.64% and 3.88%, respectively, of all SUs.

**Figure 12:**
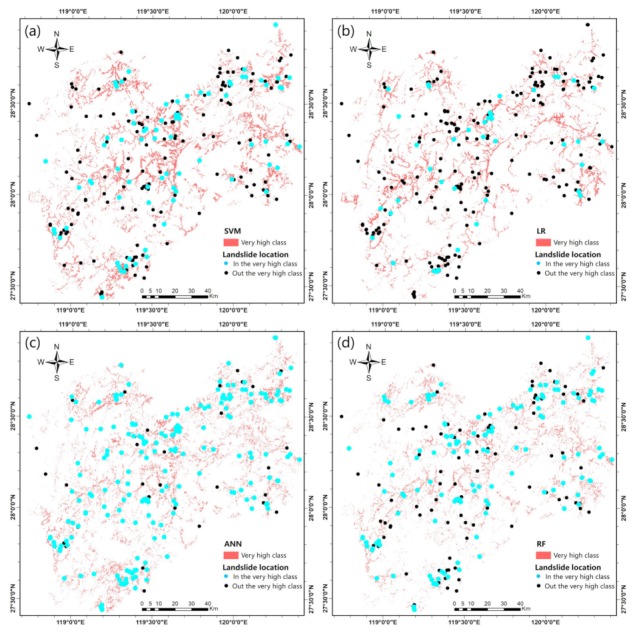
The very high susceptibility class areas of the landslide susceptibility maps: (**a**) SVM model; (**b**) LR model; (**c**) ANN model; and (**d**) RF model.

The class-specific accuracy indicates the classification accuracy of a landslide model, especially for the very high susceptibility class, meaning that the model has the ability to detect areas with a very high susceptibility, mainly including previous landslides [78]. For the class-specific accuracy of the very high susceptibility class, the ANN model achieved the highest percentage with a value of 0.5014%, followed by the RF (0.4767%), SVM (0.1779%), and LR (0.1381%) models. In addition, Figure 12 displays the very high susceptibility class areas generated by the four machine learning models. With the distribution of landslide locations, we found that the SVM and LR models generated more very high susceptibility class areas than did the other two models, and several landslides were not located in areas with a very high susceptibility. The SVM model generated the greatest number of very high susceptibility class areas, while the LR model identified the fewest landslides, indicating that both models suffer from poor classification ability. The RF model generated the fewest very high susceptibility class areas and identified more landslides. The greatest numbers of landslides were located in areas with a very high susceptibility in the ANN model, indicating that the ANN model performed the best among all four models and generated the most reasonable landslide susceptibility map.

## 4. Discussion

Machine learning methods have recently been applied in landslide susceptibility mapping. However, although previous studies focused on using different machine leaning methods to improve the landslide susceptibility model performance and achieved good results, the sample sizes utilized for the modeling were not sufficient for machine learning. Therefore, we used SMOTE to obtain sufficient samples for four machine learning methods—SVM, LR, ANN, and RF models—to produce landslide susceptibility maps.

The data situation in this study could be considered as a PU (positive and unlabeled) problem, in which only positive data (landslide data) and unlabeled data are available. We adopted a common method that treats the unlabeled data as highly noisy negative data (non-landslide data); then, we randomly collected the same number of negative data, which were then added to the testing landslide dataset and each training landslide dataset for subsequent modeling. Non-landslide areas account for most of a given city, and thus, the number of unlabeled samples in the study area is very large; therefore, the noise of the negative data could be restricted, and the final results suggest that this method worked well. PCC, PCA, and NRS methods were used to determine the most important factors for landslides. The results indicate that the slope and PCI were two of the most significant factors in the study area. These results are reasonable because the slope is well known as the most important factor affecting the landslide susceptibility [79,80], while rainfall is heavy throughout the study area and can undermine the stabilities of slopes, leading to landslides.

The accuracy, kappa index, and AUC were used to evaluate and compare the performances of the landslide susceptibility models using the real training dataset and 29 different sets of training data generated by SMOTE. The results reveal that the fitting and predictive abilities of the four models improved as the sample size increased, and there was a significant improvement between the first and 30th training datasets, proving that the use of SMOTE to improve the performances of machine learning methods for landslide susceptibility mapping in this study is reasonable. The reason for the differences between the fitting and predictive abilities of the four models is that the fitting accuracy measures how the predictive values from the landslide susceptibility models fit the training dataset, while the prediction accuracy can explain the degree of fit of a landslide model over the testing data that did not participate in the model training, thereby reflecting the generalization ability. As a rule, the fitting accuracy is better than predictive accuracy, and the relationship between the fitting performance and predictive performance is not proportional.

In terms of the predictive ability, the ANN model was revealed to be the best: as the training data continued to increase, its accuracy tended to improve. The reason for this improvement is that many parameters are present within the neural network that require a large quantity of sample data to be trained. The curves of the accuracy, kappa index, and AUC show that the SVM model performed better than the RF model before reaching the multiple of 15; after 15, however, the SVM model was surpassed by the RF model. This phenomenon can be explained by the fact that the SVM model has an advantage when using small sample sizes based on its use of a kernel function to map multivariate data onto a higher-dimensional space, while the RF model uses an ensemble learning method that operates by constructing a multitude of decision trees, and thus, it needs more samples to perform better. The results also depict that the LR model showed only a small improvement with an increase in the number of samples and had the lowest predictive capability among all the landslide models. This indicates that the SMOTE method cannot improve the LR model well and that LR is not as effective for landslide susceptibility assessment compared with other methods presented in this work. These results arose because the LR model is linear in nature, and this algorithm performs well only when the data are linearly separable. Although the LR model could utilize the gradient descent algorithm with sufficient training samples to update the model, the improvement would be very minor due to its poor performance with multivariate data, such as landslide data. The limitations of LR have been optimized by the generalized additive model (GAM), which replaces the parameters of equation (8) with nonlinear expressions. This feature can better model complex geographical problems such as landslides. The GAM has also achieved good results in landslide problems [81,82]; hence, we will apply SMOTE to the GAM and other nonlinear models in the future to determine whether the model performance can be improved. Additionally, we found that the final accuracies of the ANN (91.43%), RF (88.6%), and SVM (86.5%) models were very close; therefore, we used the kappa index to test their consistency. The results are shown in Table 4. Very high agreement was observed between the RF and ANN models (kappa index = 0.7), while the SVM model showed a low consistency with the other three models (kappa index < 0.5). This result is similar to the conditions of the accuracy. However, the consistency between the RF and SVM models was relatively low (kappa index = 0.55) considering the accuracies of both models. Hence, both models offer correct predictions for different areas.

**Figure 13:**
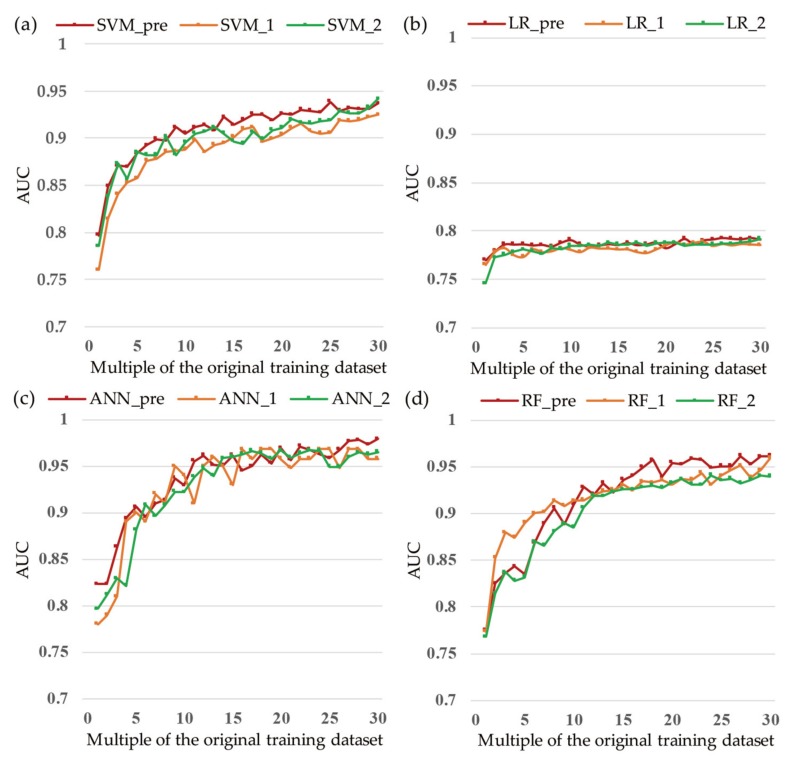
Different segmentation comparisons for several models using validation with different datasets (_pre means the previous segmentation and _1 and _2 mean the two different segmentations). (**a**) SVM, (**b**) LR, (**c**) ANN, and (**d**) RF.

The oscillations in the results shown in Figure 9 and Figure 10 can be due to two reasons. Firstly, the data substantially influence the models. In general, the quality of data rises as the size of the training dataset increases. However, there is no guarantee that a larger training dataset will be more valuable than a smaller one, due to randomness in the computations of SMOTE (used in 2–30 training datasets). The second reason could be the randomness in constructing the models. For instance, random initialization was adopted in this study to set the weights in the gradient descent algorithm (used in the LR and ANN models). This strategy creates uncertainty about the results. The partitioned landslide SUs between the training dataset and testing dataset were randomly selected in the study area. To determine whether different segmentations affected the fluctuations, we randomly selected two more different sets of training and testing data. A comparison of the results obtained with the three datasets is shown in Figure 13: different partitioning has a slight effect on the oscillations, but the trend of the AUC value has no change; an overall rise is present when the training samples numerosity is increased.

The results of this study confirm that SMOTE improved the performances of the machine learning methods for landslide susceptibility mapping. For comparison, we also considered a traditional model, namely, the frequency ratio (FR), using the same training datasets (Figure 13). The predictive AUC values of the FR in the first and last training datasets were 68.42% and 69.97%, respectively, and the improvement in the FR was 2.26%, which was lower than those of all the other models. These results show that the conventional method limited the predictive ability for landslide problems, and the improvement with an increase in the sample size was also finite compared with the improvements in the machine learning methods. The reason for this is that the techniques used in machine learning methods, including iterative computation and optimization algorithms, can reduce both the bias and the variance against base classifiers to improve their predictive capability. Moreover, these results are in agreement with those of Kadavi [83]: who found that the prediction performance of traditional model was worse than machine learning models in landslide susceptibility.

**Figure 14:**
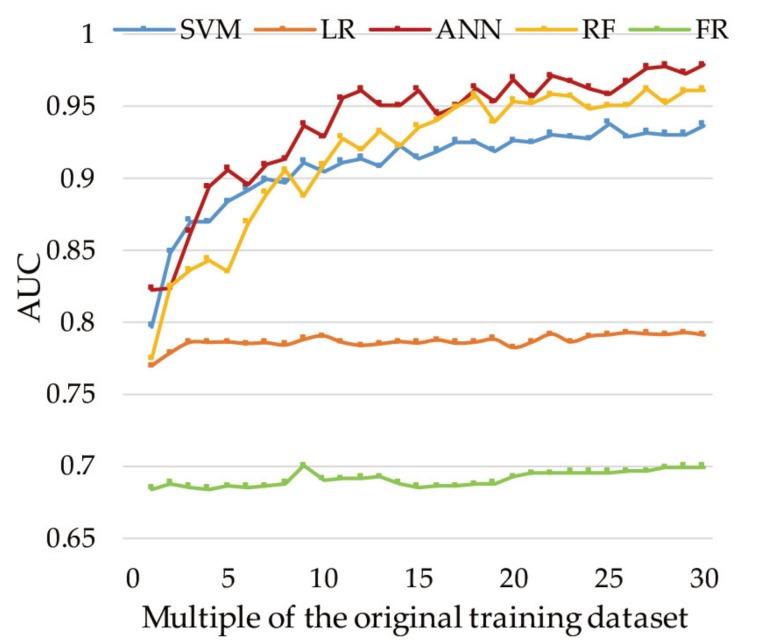
AUC of predictive performance for the RF model with different training datasets.

To discover the effect of each landslide-causing factor on the predictive abilities of the four machine learning models, we reduced each of the 12 factors in turn to retrain the models using the original landslide training data and testing data. The AUC was used to evaluate the models, the results of which are shown in Table 5. The NDVI had the greatest influence on the accuracies of the four models. Without the slope, land use, and PCI the predictive abilities of the SVM, ANN, and RF models were reduced by at least 11.7%. The TST had the least impact on the LR model, reducing the accuracy by 9.1% (Figure 14). This indicates that each landslide-causing factor, especially those extracted from remote sensing data, has a significant influence on the improvement of each model. As the mechanism responsible for landslide disasters is not yet fully understood, more reasonable landslide-causing factors and landslide samples could guarantee the predictive abilities of machine learning models for landslide susceptibility mapping.

The landslide susceptibility maps were generated for the study area by the four machine learning methods and divided into five classes by an equal interval classification. The class-specific accuracy was used to facilitate an evaluation and a comparison of the maps. The results indicate that the ANN generated a more reasonable area of very high susceptibility and identified more landslides than did the other models. This result is in agreement with the results of model evaluations, and thus, this landslide susceptibility map can aid in effective policy decisions. Although this study improved the predictive abilities of machine learning methods for landslide susceptibility mapping, certain limitations should be addressed in future work. For example, some noise is present within the non-landslide samples, and the SMOTE method does not incorporate the impact of the geographic location. Moreover, the training and application of machine learning models are very time consuming.

## 5. Conclusions

Landslide susceptibility mapping is a useful tool for government institutions to develop landslide hazard prevention and mitigation strategies. Therefore, high-performance landslide prediction models are of paramount importance. Machine learning methods have recently been used for landslide susceptibility mapping, and they have achieved better performance than traditional methods. The performance of machine learning is often dependent on a large quantity of training data; however, the sample sizes in previous studies have obviously been inadequate. Furthermore, landslides are uncommon phenomena; consequently, the sample size is unlikely to be large.

The novel contribution of this study is its application of SMOTE to oversample the landslide data to obtain a sufficient training dataset for machine learning models for landslide susceptibility mapping. In addition, we selected SUs as the mapping units due to the physical meaning of the terrain unit. The NRS method was adopted to reduce the attributes of landslide-causing factors derived from topographic and geological data and satellite images. Four machine learning models—SVM, LR, ANN, and RF—were evaluated and compared for landslide susceptibility assessments using different training dataset sizes (two to thirty times the original data). The results showed that the four models performed better with the increased dataset sizes than they did with the original training data. The RF model improved the most (AUC increased by 24.12%), followed by the ANN (AUC increased by 18.94%), SVM (AUC increased by 17.77%), and LR (AUC increased by 3.00%) models. In particular, the LR model improved very slowly and only to a limited extent because it is essentially a linear classifier and exhibits poor performance with multivariate or nonlinear data. Furthermore, the ANN model achieved the best performance for landslide susceptibility mapping (accuracy = 91.43%, kappa index = 0.83, AUC = 0.98). Finally, the four models that performed the best from each of the four types of machine learning methods were applied to generate landslide susceptibility maps of Lishui City in Zhejiang Province, China.

This work improved the predictive ability of machine learning methods in landslide susceptibility mapping by expanding the training dataset using SMOTE, and the resulting landslide susceptibility maps could help decision makers to plan more effectively. 

## Figures and Tables

**Table 1:** Landslide conditioning factors in this study.

Category	Conditioning Factors	Type	Range
Predisposing factors	Slope (°)	Continuous	(0, 80.39)
	Elevation (km)	Continuous	(0, 1.92)
	Aspect	Categorical	Flat, North, West, South, Southeast, East, Northwest, Southwest, Northeast
	Curvature	Continuous	(−26.54, 39.78)
	Plan curvature	Continuous	(−17.76, 16.51)
	Profile curvature	Continuous	(−25.50, 19.98)
	Distance to faults (km)	Continuous	(0, 1.85)
	Distance to rivers (km)	Continuous	(0, 3.22)
	Distance to roads (km)	Continuous	(0, 5.95)
	Land use	Categorical	Roads, Structures, Water, Planting Land, Desert and bare land, Forest and grass, Artificial heap, House building
	Engineering geological type	Categorical	Group 1, Group 2, Group 3, Group 4, Group 5, Group 6, Group 7, Group 8, Group 9, Group 10
	NDVI	Continuous	(−1, 1)
	TWI	Continuous	(0.74, 46.81)
	TRI	Continuous	(0, 67.52)
	TST	Continuous	(0, 100)
Triggering factors	Earthquake influence	Continuous	(0, 0.44)
	Annual precipitation in the wet season (mm)	Continuous	(1020.45, 1417.50)
	Annual precipitation in the dry season (mm)	Continuous	(428.81, 588.96)
	Annual precipitation (mm)	Continuous	(1459.57, 1990.30)
	Annual torrential rain days (day)	Continuous	(13.23, 20.12)

**Table 2:** Principal component analysis (PCA) results.

Index	PC1	PC2	PC3	PC4
Explained variance (%)	90.434	5.784	3.780	0.002
Cumulative explained variance (%)	90.434	96.219	99.998	100.000
Eigenvalues	3.617	0.231	0.151	6.110 × 10^−5^

**Table 3:** The class-specific accuracies of different models (NOL: number of landslides; NOS: number of SUs; PLS: percentage of landslides to SUs (class-specific accuracy); PSS: percentage of SUs to all SUs in the study area).

Model	Index	Very Low	Low	Moderate	High	Very High
SVM	NOL	20	15	46	91	116
	NOS	534,060	123,388	97,210	94,599	65,218
	PLS (%)	0.0037	0.0122	0.0473	0.0962	0.1779
	PSS (%)	58.4	13.49	10.63	10.34	7.14
LR	NOL	17	24	81	94	72
	NOS	294,866	210,885	197,711	158,891	52,122
	PLS (%)	0.0058	0.0114	0.041	0.0592	0.1381
	PSS (%)	32.24	23.06	21.62	17.38	5.7
ANN	NOL	3	3	9	36	237
	NOS	607,082	146,554	72,170	41,402	47,267
	PLS (%)	0.0005	0.002	0.0125	0.087	0.5014
	PSS (%)	66.39	16.03	7.89	4.53	5.16
RF	NOL	1	22	27	69	169
	NOS	542,796	193,917	99,872	42,435	35,455
	PLS (%)	0.0002	0.0113	0.027	0.1626	0.4767
	PSS (%)	59.36	21.21	10.92	4.64	3.87

**Table 4:** The consistency among the accuracies of the models.

Model	SVM	LR	ANN	RF
SVM	1	0.54	0.57	0.55
LR	0.54	1	0.43	0.49
ANN	0.57	0.43	1	0.7
RF	0.55	0.49	0.7	1

**Table 5:** The AUC values of the models with different reduced landslide-causing factors.

Reduced Factor	SVM	LR	ANN	RF
None	0.79	0.77	0.82	0.77
NDVI	0.63	0.63	0.70	0.62
Slope	0.67	0.68	0.72	0.68
Land use	0.68	0.68	0.72	0.67
PCI	0.68	0.69	0.72	0.66
Elevation	0.68	0.68	0.74	0.65
Distance to Rivers	0.68	0.68	0.72	0.68
Aspect	0.69	0.66	0.71	0.66
Distance to Faults	0.69	0.67	0.72	0.68
Engineering geological type	0.69	0.68	0.74	0.67
Distance to Roads	0.69	0.69	0.76	0.66
Profile curvature	0.70	0.67	0.72	0.66
TST	0.71	0.70	0.75	0.66
